# Lecture, Introductory to the Course upon the Microscopical and Comparative Anatomy of the Teeth, Delivered in the Baltimore College of Dental Surgery

**Published:** 1857-01

**Authors:** Christopher Johnston

**Affiliations:** Baltimore.


AMERICAN JOURNAL

OF

DENTAL SCIENCE.

Vol. VII. NEW SERIES—JANUARY, 1857. No. 1.

### ORIGINAL COMMUNICATIONS.

### ARTICLE I.

*Lecture, Introductory to the Course upon the Microscopical and Comparative Anatomy of the Teeth, delivered in the Baltimore College of Dental Surgery.* By CHRISTOPHER JOHNSTON, M. D., Baltimore.

GENTLEMEN: I am aware that the subjects I shall have the honor of treating this winter are not altogether novelties to you; but it shall be my pleasure to present, in the course upon which we are entering, a *connected* and *comprehensive* view of the microscopical and comparative anatomy of the teeth, and of the soft parts having intimate relations with them.

The present condition of the scientific portion of your studies renders a casual and brief attention to these branches both uninviting and unsatisfactory; wherefore the honorable faculty of the Baltimore College of Dental Surgery, taking the initiative in this country, have given to these branches the consequence which their importance demands, and open to *you* a new field of entertaining and profitable investigation.

Before entering seriously upon our work, I desire to occupy you during a portion of this hour with the *microscope*, the instrument which increases the capacity of the eye for viewing small objects; and to conclude with an exposition of the uses and advantages of the study of comparative anatomy.

The latter part of the sixteenth century was distinguished by three remarkable discoveries, which made their impress not only on the age that witnessed them, but on all succeeding ages; which have guided the intelligence of man in the investigation of the phenomena of life, and the explanation of the minutest recesses and particles of organized matter, and extended his inspired gaze almost prophetically into regions of the glorious universe which the *imagination* had claimed as her own peculiar sphere. These great discoveries were the *circulation of the blood*, the *microscope*, and the *telescope;* but like other inventions belonged hardly to one man or a single epoch, although the entire merit of originality was given to those men who first made the truth to be recognised and accepted. Hippocrates knew that the blood was not stagnant in the veins—Galen was aware that two sorts of blood existed in the body, that there were two sorts of blood vessels, and that they both communicated with each other and with the heart—he was on the threshold of one of the greatest of discoveries, yet he failed to advance a single and final step which would have placed the wreath upon his brow. But at length Michael Servetus, a Spaniard, armed with all the knowledge of his predecessors, proclaimed the circulation of the blood in a theological tract ("Christianismi Restitutio," &c.,) in 1553, for which tract, however, he was burned at the stake by Calvin, in Geneva, a few months after its publication.

The knowledge of optical glasses, or *lenses*, which form the essential part of both optical instruments, did not first belong to those cunning men who sketched out the earliest rude microscope and telescope; for, setting aside the hoary antiquity of Chinese astronomy, it is certain that more than five centuries before Christ, Pythagorus was acquainted with lenses; that one hundred years later, Aristophanes, in his comedy of "The

Clouds," makes distinct mention of a "burning glass;" that Aristotle, 354 years A. C. speaks of means to assist the vision of near-sighted and far-sighted persons; that Seneca and Pliny, at the commencement of the christian era were perfectly cognizant of the use of refracting spheres of glass, as was also Roger Bacon in the 13th century; and finally, that Claudius Ptolemæus, of Alexandria, (Egypt,) who lived in the *second* century, is represented in an illuminated manuscript of the 13th century, as gazing at the stars through a tube composed of several pieces. Nevertheless, the invention of the telescope is claimed to have been made at the end of the 16th century, that of spectacles to have followed it early in the 17th, in consequence of two children of an optician amusing themselves by looking through lenses at a weathercock; while the first practical microscope is attributed to one Zacharias Jansen, of Middleburg, in Holland, in the year 1590, which is also the date assigned to the invention of the telescope. To the learned, these things were, most probably, no novelty; but the real merit of the so-called inventors, consisted in their recognition of the true principle of refracting media, and their knowledge of the means whereby lenses differently arranged might be applied to various practical uses. It is true that the earliest optical instruments were of extremely simple construction—the eye piece of Newton's telescope and the single lens microscope of Leeuwenhock, in the 17th century, are examples of them. Yet the eye of the observer, in these instances partially supplied the defects of his glasses, which passed from his hand not without having undergone some modification of improvement. With his telescope, Newton scanned the heavens and singled out particular stars in the milky way: and with his simple microscope, Leeuwenhock, with untiring industry, studied bone and muscle, blood-corpuscle and the circulation of the blood, and nerves and vegetable tissues, and even the extremely minute spermatic particles.

Now, I hold it to be extremely useful for me to set before you the examples of such men as those I have named: their success depended less upon the instruments they employed than upon their determination to accomplish a great work, for their zeal comprehended in its scope as well the instruments they used for investigation as the objects upon which the latter were directed. Their heads were right, and their hearts were right—and in their admiration of the beauty of form, or color, or motion, they worshiped a divine omnipotence, in the minutest animated particle, and in the rythmic vibrations of the orbit of the whirling planet as it swings in space like "a mighty pendulum marking the seconds of eternity."

Lest you might attach undue importance to the complications of the microscope, or be embarrassed at the onset with mystical "laws" of optics, I intend, in brief and simple language, to make you acquainted with that which is *essential* in the construction of the instrument not less than with the manner in which it assists the natural vision.

A *simple microscope* consists of one single lens, or of several lenses having the effect of one lens—set in a frame for convenience of manipulation. This was the earliest form which the microscope assumed.

A *compound microscope* is composed of several lenses acting independently as well as conjointly, and arranged at certain distances from each other along a tube, which, as a setting, offers the advantages of security, and mobility in the direction of the axis or line passing through the centre of all the lenses.

A microscope shows us a magnified picture of a small object, and it does nothing more; but it may present to our retina an image perfectly or imperfectly defined according as its lenses—or really itself in these its essential parts—are perfectly or imperfectly constituted, fashioned or adjusted. If the lens be, then, the microscope, we have only to understand the manner in which the former acts upon rays of light, in order to comprehend the mode of action of all microscopes, whether *simple*, or of a single element, or *compound*, or of many elements.

When light passes through *space* it pursues a straight line in its course, if it next transverse *air*, the rays are bent, because air has a certain density, whereas space or ether has none; if now it enter glass or other transparent substance or medium, then the rays are still farther bent, for the reason that water, or crystal or glass has a greater density than the atmosphere. Finally, if the rays again pass through atmospheric air into space, they recover completely their original direction.

A lens, such as we have to do with, is a transparent dense body, whether of glass, of ruby or of diamond, which bends or refracts all rays of light traversing it towards a single point or *focus;* and this property of focalising is entirely owing to its form, which is curved like a portion of a sphere on one side and flat on the other; or else it may be so curved, or *convex*, on both sides, becoming thereby more powerful.

The lower and smallest lenses of the compound microscope are known as the *objectives*, because they first magnify the object which lies immediately beneath them; while the uppermost lenses in the tube form the *ocular* or *eye-piece*, so called for the reason that it magnifies the picture of the object first formed in the tube by the *objective*, and presents it to the eye. Consequently, the instrument we are considering twice magnifies an object before the eye takes cognizance of it: and, furthermore, we see an object magnified because its *image* makes with the eye, by reason of the bending of the rays of light which proceed through the lenses of the microscope to the organ of vision, the same angle which a real object would make were it 10, 20, 100 times as great. Therefore, you will perceive, that the size of an object is measured by our sense by the angle at which two lines meet the eye when they proceed from the edges of that object. It is called the *visual angle;* and it increases, or in other words the object is magnified, whenever the latter approaches the eye, or the lenses of the microscope bend the rays issuing from the object and cause them to appear *as if* they proceeded from a nearer or a larger object.

Now, gentlemen, I hope that you are not wearied with this brief exposition of the theory of the instrument that is to guide us in our future studies; but if you have followed me in the simplest explanations I have been able to give, you must have arrived at these very truthful and remarkable results, namely, that the microscope magnifies objects by enabling the observer to inspect them *as if* they were very near the eye whereby their visual angle is increased—in other words, it exaggerates near-sightedness. And again, as the microscope presents the *image* only of an object to the eye, vision assisted by that instrument is just what it was before, "the art of seeing things that are themselves invisible."

Having thus succinctly told you what the microscope *is*, it becomes me, in the next place to inform you what it *does*.

In its performance, two things are accomplished: in the first place, objects are rendered distinctly visible which, on account of their small size are, under ordinary circumstances, imperfectly or indistinctly seen; and in the second, the eye is made acquainted with a vast number of objects and parts or details of objects, which wholly escape the unassisted vision. Those of the latter class are popularly denominated *microscopic objects*, but you may be not a little surprised to learn how diminutive are their dimensions. Each of you, with attention, can readily discern spaces between lines measuring only the $\frac{1}{600}$ of an inch in breadth—if the object reflect light, as a particle of gold, you may still perceive it, although its width do not exceed the $\frac{1}{1100}$th of an inch—if it be black upon white, its size may be still further reduced to the $\frac{1}{2700}$th of an inch without becoming invisible; and finally, which of you has not seen the brightly illuminated and fairy like gossamer as it floats upon the summer air, or beheld the busy glittering of "motes that people the sunbeam," when a necklace of 5000 such particles would hardly be measured by a single inch. Beyond this limit objects are lost to the unaided eye; and it is here that the microscope "takes up its wondrous tale."

But although the marvellous organ of vision can grasp such tiny molecules, it is superfluous to observe, that beyond a knowledge of position and approximative size we learn nothing, and we remain ignorant of form, constitution and nature. It is natural that we should desire to know all the characters of objects that tantalize our gaze, independently of the laudable anxiety which honest students entertain for fathoming the mysteries of organization. But what happens when we arm our eye with an instrument which unveils so many secrets? Is our curiosity entirely gratified? Is our anxiety perfectly satisfied? By no means. The difficulties overcome take flight and we rejoice that they vanish; but they quit our neighborhood to settle at a greater distance, and eventually we realize the fatal truth that the limits of knowledge are not destroyed but only farther removed.

Nevertheless, the circle of attainment is widened around us—and while we thought to be as gods in the allotted garden of intellectual paradise, we find that the microscope opens to us new fields of labor, of wonder and delight. We make an anatomical analysis of man's body, yet the same means we employ betray to our admiring sense the complex organization of the tiniest animalcule of a drop of water—the green filaments that paint the rock, bared by the ebbing tide, become aquatic trees, amid whose branches the most varied creatures wanton like butterflies: we call by name myriads of beings of the vegetable and animal kingdoms of whose very existence we hardly dreamed; we can almost distinguish the lowest and simplest animal from the most rudimentary vegetable formation; and with polarized light we discover the aurora that lurks in the very smallest pellucid crystal.

If, gentlemen, you bear in mind that it is not the eye which sees, but the intelligence *by* the eye, you will readily perceive that microscopical studies have nothing peculiar in themselves further than they are an extension of studies which the unassisted organ of vision is able to pursue. The objects comprehended in them both are the same; and the human mind is called upon to discriminate in the same manner between reality and similitude, or what *is* and what *appears* to be, whether it be exercised within the natural limits of our senses upon the mastodon or the mouse, or whether, by the assistance of the microscope those limits being farther removed, it is directed towards the mosquito or the monad.

After all, are we much the wiser for all that the microscope has revealed? Is our practice advantageously modified in consequence of our new acquisition? The answer to these interrogatories brings me to the second part of my subject.

It is hardly necessary to remind you, that from a single fact we are unable to make a generalization; and it must be equally evident to you, that a generalization, or its expression a *law*, approached nearer to the truth than another law when the former is the resultant of a greater number of facts than the latter. Now all phenomena are wonders and novelties to the individual whose information is limited to a knowledge of a few of them; and whatever pleasure he may enjoy from an acquaintance with them he is utterly incompetent to appreciate their importance or to render their study profitable to others, *because* he cannot localize the facts or phenomena, or assert their connection with other truths which tend to constitute a harmonious whole. To know that a muscle contracts, that in man it exercises *force*, is something; but this knowledge has a circumscribed usefulness, unless accompanied with an acquaintance with the structure of muscle, its mode of attachment to the levers of the body, the manner in which its minute contractility is called into operation, its electrical condition during the periods of contraction and relaxation, its connection with nerves and blood vessels, its nutritition and the conditions under which its exhausted energy is restored. But the task does not end here, for it is necessary to know that there are in man two sorts of contractile tissue, that the arrangement of its blood vessels presents peculiarities which involve an acquaintance with the rest of the sanguiferous system and then of the blood, that the nerve-filaments *arouse* an inherent energy, that these possess or convey a force which must be recognized, &c., and thus the circle widens. It is true that without all this or with it we are equally satisfied that a muscle contracts, but we derive certain and positive advantage from knowing all the conditions upon which depends the integrity of the muscle, as well as those attending the phenomenon of contraction.

Apart from variety we are accustomed to assign to *man* the highest place in the scale of creation, and to determine the position of other animals according to their departure from man, the assumed type or measure of perfection: and this is strictly the province of comparative or philosophical anatomy. But it must be borne in mind that man owes his high position to the aggregate of the mechanism of his body, of its various functions and to the variety of the faculties of his mind, and not to the absolute perfection of every particular organ or of each especial function. The lion scents his prey, the eagle espies his quarry, the dog catches the faintest sound from a distance, with far more precision than man can smell, or see or hear; nevertheless the development of the noblest part, the nervous system in general, positively surpasses that of every other creature; and as his faculties and capacities stand in constant relation to the degree of perfection of the nervous centers, and as it is very certain that all the organs which execute, which are its subordinate instruments, have a dependent and commensurate development, we place *man* at the head of creation, as consequences of his admirable organization he walks erect amongst the creeping beasts; he alone of all animals possesses that requisite organ, the *hand*, with an opposable thumb; and his chin projects, because, unlike the "beasts that perish," his jaws do not seize the grass or devour a palpitating victim, but they mediately receive the food which man's mastery over *fire* enables him to adapt to his wants.

I have explained the end or object of "philosophical or comparative anatomy, which is purely a generalization, and is related to human anatomy in its dogmatic part only, in that which has reference to its plan of teaching and study." Allow me, in the next place, to expose the objects and principles of the anatomy of animals, which is popularly confounded with comparative anatomy; indeed, the opinion has the practical support of a small number of men of science. "Comparative anatomy (says Giraldes) seeks to find, in general expressions, the type of being, the type of any organ whatever, and to follow the modifications and degradations which this being, this organ experiences in all the animal series." The anatomy of animals, gentlemen, has, on the other hand, the same object as human anatomy; and this is, the study and knowledge of tissues and organs. General anatomy pursues the investigation of tissues and organs still further, and seeks to discover, by every available means, such as injections, macerations and the microscope, the visible elements which build them up; and to follow the latter through the different phases of their development. It affects to know their physical and chemical properties, and to recognize the changes wrought in them by death.

Thus you cannot fail to discover that human anatomy may be successfully studied without recourse being had to dissections of the lower animals, for elements, tissues and organs, with a varying modicum of intelligence, constitute an individual creature, complete and perfect of its kind. And I may say the same with regard to animated anatomy, which is physiology; for however closely certain processes or functions in a dog *resemble* corresponding actions in the human subject, we are far from establishing *identity* of operation; and we would be wide of the truth if we admitted more than *analogy.* The anatomy of the vascular system of a fish might lead us to study with greater effect that peculiar transitory condition of the same system in the human embryo, which most nearly resembles it; but we have no right to assume the perfect applicability of *any* fact in one creature to another—such presupposition being entirely erroneous. And again, if by injecting a solution of prussiate of potash into one jugular vein of a dog, and marking the interval which elapses before its reappearance in the blood of the other jugular vein, (having made the circuit of the body,) we suppose the circulation of the blood to be accomplished in twenty seconds, we are not in consequence permitted to do more than assign approximative limits to the velocity of the blood in man; for even in the dog, which is of lesser size, a different salt used in the experiment gives a different result, and this again varies with the condition of plethora or anemia of the animal, with the amount of pain inflicted and of terror experienced. But herein does a knowledge of the anatomy of animals assist the student of human anatomy—it is useful in pathological anatomy and the study of forms, it is very serviceable in the study of structure and indispensable in the study of development.

I have stated that general *human* anatomy strives for an intimate acquaintance with elements, tissues and organs. But in *man*, these are always difficult to obtain in the normal conditions—the harder tissues oppose complexity of structure to observation, and the softer tissues readily undergo cadaveric change before they can be procured for investigation; wherefore it is oftentimes necessary to resort to dissection of animals, which are rarely effected with disease, and which may be submitted to examination immediately after their immolation. And for a still further reason are the organs of animals useful in the study of corresponding parts of the human subject—they are frequently less complicated, and in the lowest classes are reduced to their simplest expression, so that analogous elements may be met with freed from many of the complications which perplex the student; but furthermore, as in the serial degradation parts of subordinate importance are successively eliminated, the essential nature of those which remain may be clearly made out by reason of their constancy. For it is a proverb in anatomy as well as experimental physiology, that "what is not constant is not essential."

You will have now no difficulty in understanding the true end or aim of that branch of study upon which we are about to enter. It is in part philosophical, that is in so far as concerns our recognition of the type of particular organs and the assistance they give in the determination of the type of the creature—but it is also highly practical, as relating to the origin and development of those beautiful organs *the teeth;* their structure and the fitness of the means, or their elements to accomplish particular ends; their simplification in the animated chain, both in elementary constitution and form as we recede from the golden link of humanity in accordance with the successive restriction of their function; and the seal set upon their constituents in the molecular death which undermines their fair fabric.

In learning all this you become better men, because your hearts must be lifted up in admiration of the cunning handiwork of the great artificer of the universe; and you are better fitted to be apprentices of divine nature and imitate her works, and to overcome the destroyer, who follows fast in her footsteps, ever ready to sever the ties of life and crumble all beauty into dust.

Is the eye alone sufficient to unravel the web of the tissues or to single out the particular elementary molecules which constitute them? Can the unassisted vision seize upon those details of structure on which depend the force and permanency of the organs of our wonderful body? Can the natural sight penetrate the hazy dawn of organization or discover the mysterious workings of disorganization and death?

Not so, gentlemen, in these particularities the mind must have ever remained unsatisfied but for the microscope, that instrument which gives a "local habitation and a name" to things before unseen, which surpasses the limits of our sense, and which foreshadows a horizon of knowledge of wondrous extent.

### ARTICLE II.

*Papers on Dental Pathology.* By S. JAMES A. SALTER, M. B., F. L. S.*

